# High-Fat Diets Fed during Pregnancy Cause Changes to Pancreatic Tissue DNA Methylation and Protein Expression in the Offspring: A Multi-Omics Approach

**DOI:** 10.3390/ijms25137317

**Published:** 2024-07-03

**Authors:** Lindsey Eileen, Maria Peterson

**Affiliations:** Department of Fisheries, Veterinary, and Animal Science, University of Rhode Island, 45 Upper College Rd., Kingston, RI 02881, USA; lindsey_eaton@uri.edu

**Keywords:** pancreas, developmental programming, DNA methylation

## Abstract

Maternal obesity, caused by diets rich in fats and sugars during pregnancy, can predispose offspring to metabolic diseases such as diabetes. We hypothesized that obesity during pregnancy leads to increased DNA methylation and reduced protein expression in factors regulating β-cell function and apoptosis. Female C57BL/6J mice were fed a high-fat diet (HFD; 42% fat content; n = 3) or a control diet (CON; 16% fat content; n = 3) for fourteen weeks before and during pregnancy. Offspring were euthanized at 8 weeks and pancreatic tissue was collected. Isolated DNA was analyzed using whole-genome bisulfite sequencing. Protein expression was quantified using LC–MS. No significant differences in body weight were observed between HFD and control pups (*p* = 0.10). Whole-genome bisulfite sequencing identified 91,703 and 88,415 differentially methylated regions (DMRs) in CON vs. HFD male and female offspring. A total of 34 and 4 proteins were determined to have changes in expression that correlated with changes in DNA methylation in CON vs. HFD males and females, respectively. The majority of these factors were grouped into the metabolic function category via pathway analyses. This study illustrates the complex relationship between epigenetics, diet, and sex-specific responses, therefore offering insights into potential therapeutic targets and areas for further research.

## 1. Introduction

Obesity is a significant global health issue with 31.1% and 42.5% of people in the world considered to be overweight and/or obese, respectively [1]. This subset of the population is expected to increase to 50% in the next 10 years [2]. Furthermore, in developed countries, obesogenic diets with high-sugar and high-fat concentrations are common and are contributing to the obesity epidemic [2]. Unsurprisingly, obesity in pregnant women is also on the rise with 29% of women being considered obese prior to pregnancy [3] and 48% of women in the United States gaining more than the recommended amount of weight during pregnancy [1]. Maternal obesity during pregnancy can not only increase placental glucose transfers but can also impact the expression of hormone and cytokine receptors [4]. This is problematic, as it predisposes the offspring to the effects of developmental programming. It is well established that offspring born to obese or overweight mothers are predisposed to metabolic disorders as well as other diseases later in life [5,6,7]. For example, offspring born to obese mothers exhibit increased incidence of developing obesity themselves as well as insulin resistance, type II diabetes, renal disease, and other associated disorders in humans [7,8,9]. It has also been observed that overnutrition during pregnancy can result in islet hypertrophy and increased insulin production in fetal lambs [10,11,12]. Clear differences have been seen in glucose tolerance test responses between HFD and CON groups in sheep, with the HFD groups displaying higher circulating glucose and increased insulin levels [10].

It has also been demonstrated that these effects are not restricted to just the F1 generation. For example, obesity during pregnancy can lead to increased circulating insulin concentrations in mature F1 and F2 offspring [2,10,13]. To understand the impact of developmental programming on the pancreas, research has focused on identifying the molecular mechanisms that cause these changes in the F1 as well as subsequent generations with the hopes of developing interventions for these individuals [6,14,15]. One group of potential mechanisms that has been the focus of developmental programming research are epigenetic modifications. Epigenetic modifications regulate the availability of the DNA for transcription as well as the transcripts produced from genomic DNA, resulting in changes to gene expression [16]. DNA methylation, histone modifications, and small non-coding RNA species comprise the major types of epigenetic modifications [16]. It has been determined that the maternal environment that a fetus is exposed to, including a maternal high-fat diet, can cause changes to some if not all of these epigenetic modifications [17]. We chose to focus on DNA methylation given its integral role in the maturation and secretion of β-cells of pancreatic tissue [18]. Furthermore, when a dam is fed a diet rich in methyl donors, the agouti offspring, which are more prone to obesity, experience some recovery of their wild-type phenotype [19]. This demonstrates the implications of maternal diet on DNA methylation and offspring [19]. Research conducted in our lab group has already demonstrated that overnutrition during pregnancy results in changes to global DNA methylation patterns during late fetal development in an ovine model [12]. Specifically, increased DNA methylation occurred in male and female offspring [12]. However, significant gaps in knowledge remain as to if changes in global DNA methylation patterns can occur as a result of maternal overnutrition as well as if these changes are able to impact the expression of the corresponding proteins.

Therefore, we hypothesized that maternal obesity prior to and during pregnancy caused by ingestion of a high-fat diet would lead to increased DNA methylation patterns at key regions of the genome relating to pancreatic function. WGBS data provided us with a rich dataset to compare with other research studies as well as our own data in the hopes of distilling out key targets to focus on for future research as well as tailoring appropriate intervention strategies and treatment plans for affected individuals.

## 2. Results

### 2.1. Dam and Offspring Body Weights

**Figure 1:**
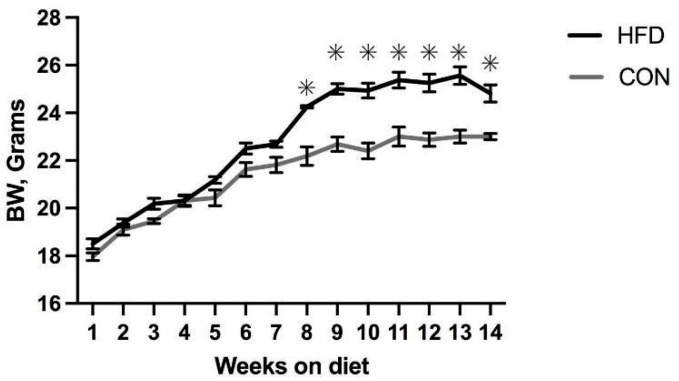
Dam body weight prior to pregnancy. * *p* ≤ 0.05 determined using Student’s *t*-test BW in female mice after 14 weeks on an HFD (n = 16) compared to 14 weeks on a CON (n = 16) diet. Significant changes in weight were observed in weeks 8–14 (*p* ≤ 0.05).

HFD mice had an average starting weight of 18.5 ± 0.42 g (g) and the CON mice had an average starting weight of 17.96 ± 0.32 g (*p* = 0.28). Over time, the average body weight of the female mice fed a control diet was 6.26% less than the female mice maintained on an HFD (21.42 ± 0.41 g vs. 22.85 ± 0.10 g; *p* = 0.01). This difference began at week eight (*p* < 0.01; HFD 24.25 ± 0.11 vs. CON 22.41 ± 0.79) (Figure 1 and Table 1) and was observed throughout week fourteen (*p* = 0.01; HFD 24.81 ± 0.73 vs. CON 23 ± 0.27).

There was no significant difference observed in offspring body weight (BW) between the two treatment groups (*p* = 0.10). However, there was a difference between the sexes in body weight. Specifically, the average weight of the male mouse pups was greater than the female mouse pups in the HFD (Male: 19 ± 0.54 vs. Female: 16.43 ± 0.52) and CON groups (Male: 21.25 ± 1.38 vs. Female: 17.25 ± 0.31, respectively; *p* < 0.05). No difference was observed in litter size when comparing the HFD and CON litters (*p* = 0.44; Table 1).

### 2.2. DNA Methylation Analyses

**Figure 2:**
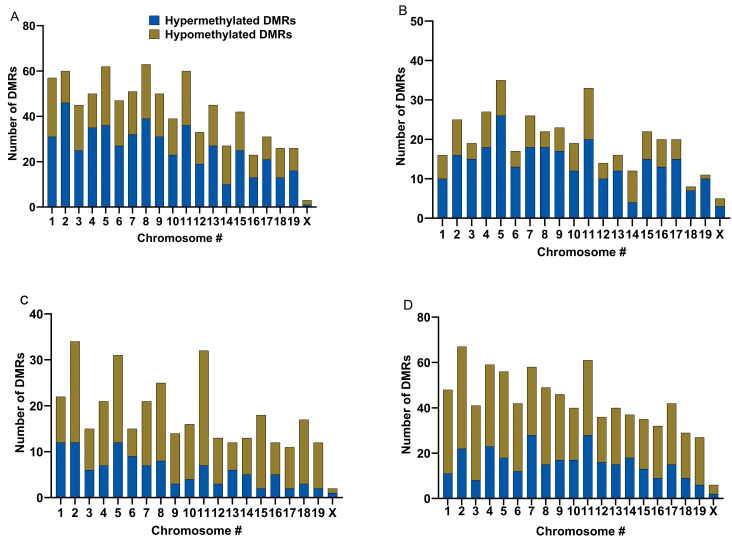
Hyper- and hypomethylation distributions within the genome of CON and HFD offspring # represents number. (**A**) # of DMRs for CON vs. HFD males; (**B**), # of DMRs for CON vs. HFD females; (**C**), # of DMRs for CON males vs. CON females; (**D**), # of DMRs for HFD males vs. HFD females. Data were determined using WGBS analysis.

**Figure 3:**
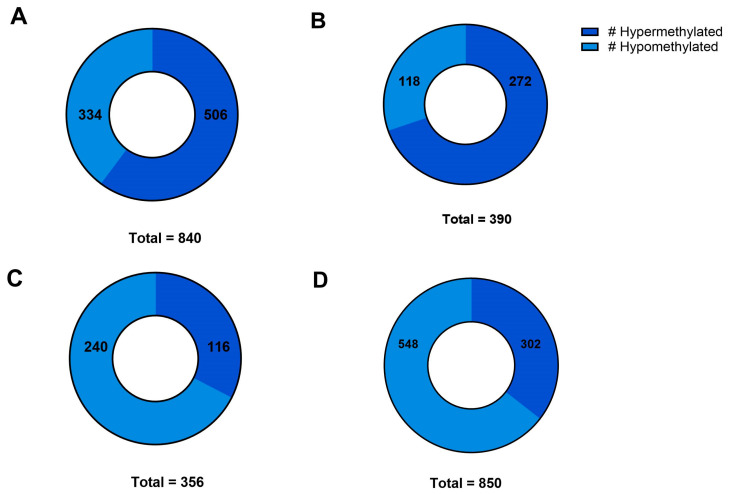
(**A**–**D**): Numbers of hypo- and hypermethylated DMRs. # represents number (**A**) # of DMRs that were hypomethylated and hypermethylated for CON vs. HFD males; (**B**) # of DMRs that were hypomethylated and hypermethylated for CON vs. HFD females; (**C**), # of DMRs that were hypomethylated and hypermethylated for CON males vs. CON females; (**D**) # of DMRs that were hypomethylated and hypermethylated for HFD males vs. HFD females. Data were determined using WGBS analysis.

**Figure 4:**
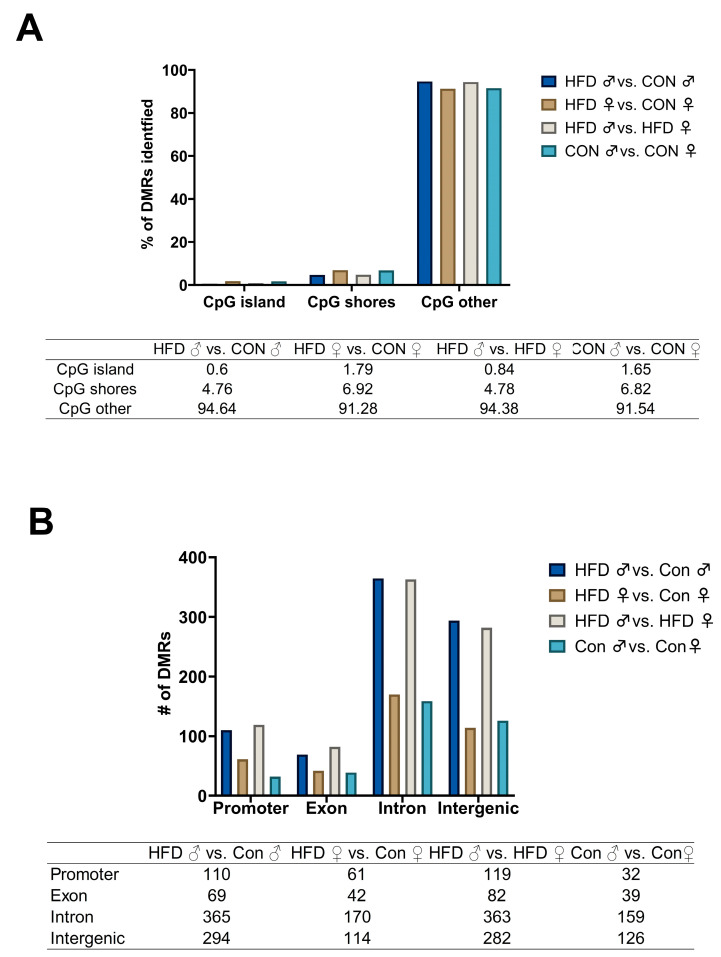
(**A**,**B**): Location and quantity of DMRs identified. # represents number (**A**) Location and quantity of DMRs within CpG islands, shores, or other; (**B**) Location and quantity of DMRs identified in cellular regions. Data were determined using WGBS analysis.

Our differential methylation analyses yielded the identification of 91,703 DMRs for CON vs. HFD males and 88,415 DMRs for CON vs. HFD females. Of these DMRs, 41,515 and 44,958 had a q-value ≤ 0.05 for CON vs. HFD males and females, respectively. Our WGBS analysis allowed us to determine the proportions of DMRs that were hypomethylated vs. hypermethylated. In CON vs. HFD males, 840 DMRs were identified in the top 25% based on the q-value, and 39.61% of those DMRs were hypomethylated, whereas 60.39% were hypermethylated (Figure 2A and Figure 3A and Table 2). Alternatively, in CON vs. HFD females, 390 DMRs were identified in the top 25%, of which 30.23% DMRs were hypomethylated while 69.76% were hypermethylated (Figure 2B and Figure 3B). When comparing sexes within the CON treatment group, 356 DMRs were identified, of which 67.41% were hypomethylated (Figure 2C and Figure 3C). In the HFD group, 850 DMRs were identified in the top 25% based on the q-value, 64% of which were hypomethylated (Figure 2D and Figure 3D). Most of the DMRs discussed above were located outside of CpG islands or shores (other: 92.96% on average; Figure 4A). CpG shores were the second most common location with an average of 5.82% of the DMRs identified being in this location (Figure 4A). A smaller fraction, 1.22% on average, of the top 25% of DMRs were located in CpG islands (Figure 4A). When looking at the genomic locations of the top 25%, the greatest proportion were found within the introns, followed by the intergenic regions (Figure 4B). A smaller fraction of the DMRs were located in exon and promoter regions of the genome (Figure 4B). These proportions were consistent across different treatment groups and sexes (Figure 4B). In CON vs. HFD males, 160 DMRs were associated with microRNAs, 1 with an enhancer region, 4 with long non-coding RNAs, 7 with SNORA, 10 with SNORDS, and 6 with small nuclear RNA species For CON vs. HFD females, 9 different DMRs were associated with non-protein coding RNA species, such as long non-coding RNAs and antisense RNAs, and 184 DMRs were associated with microRNAs.

### 2.3. Comparative Proteomics Analyses

**Figure 5:**
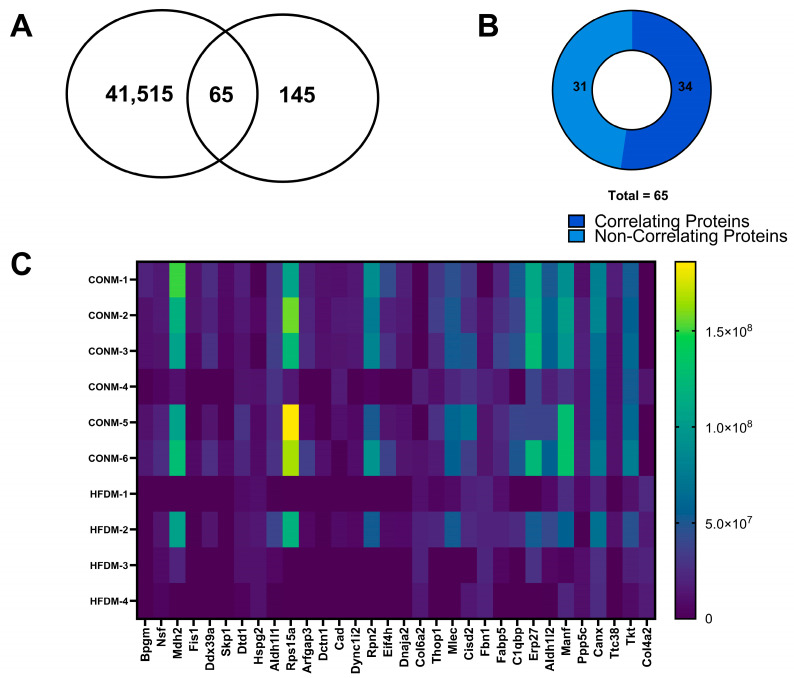
Protein quantification in relation to DMR and heatmap for correlating DMRs and proteins in the male offspring. (**A**) Number of DMRs for CON vs. HFD males compared to the number of DE proteins and those found commonly between the DMRs and DE proteins; (**B**) CON vs. HFD male DE protein correlation compared to DMRs; (**C**) DE protein expression for CON vs. HFD males. Data were determined using WGBS and LCMS analyses.

**Figure 6:**
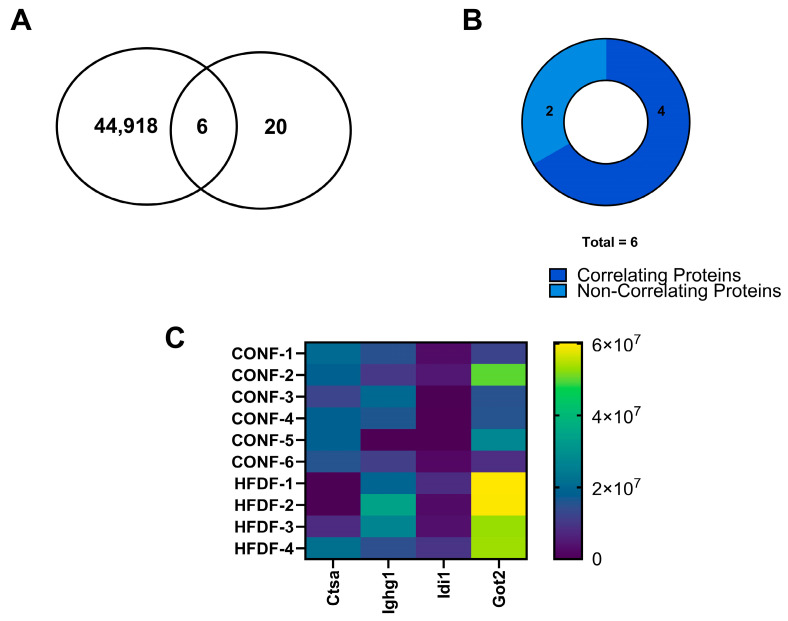
Protein quantification in relation to DMR and heatmap for correlating DMRs and proteins in the female offspring. (**A**) Number of DMRs for CON vs. HFD females compared to the number of DE proteins and those found commonly between the DMRs and DE proteins; (**B**) CON vs. HFD female DE protein correlation compared to DMR. (**C**) DE protein expression for CON vs. HFD females. Data were determined using LCMS analysis.

We were able to identify a subset of proteins in our treatment comparisons that were also identified in our DMR analyses. Specifically, 65 and 6 proteins were found to be differentially expressed that were also identified as DMRs for CON vs. HFD males and CON vs. HFD females, respectively (Figure 5A and Figure 6A). A smaller fraction of these proteins overlapped with the top 25% of DMRs identified. Specifically, this entailed three proteins (Thop1, Erp27, and Manf), when comparing CON vs. HFD males, and one protein (Isyna1), when comparing CON vs. HFD females. No similarities were found between the proteins identified and the top 25% of DMRs for the sex comparisons within a treatment group. We further analyzed the expression patterns of these proteins and their corresponding methylation changes. In CON vs. HFD males, three targets (Hspg2, Col6a2, and Col4a2) exhibited a reduction in DNA methylation with an increase in protein expression (Figure 5B,C and Table 3). Additionally, 31 targets in CON vs. HFD males showed reduced protein expression associated with increased DNA methylation of their DMRs (Figure 5B,C and Table 3). For CON vs. HFD females, reduced expression of one protein (Ctsa) was observed along with increased DNA methylation in the associated DMR (Figure 6B,C and Table 4). Conversely, three proteins (Ighg1, Idi1, and Got2) exhibited increased protein expression with reduced DNA methylation at associated DMRs (Figure 6B,C and Table 4). We were able to identify where in the genome the DMR with associated changes in protein resided. For example, in CON vs. HFD females the DMR for Ctsa spans the upstream region as well as the first intron (Table 4). As for Idi1, the DMR spans the first exon as well as a portion of the first intron (Table 4). For Ighg1 and Got2, the DMRs are located downstream from the introns and exons that code the gene (Table 4). For the CON vs. HFD males, 10 DMRs were intragenic and spanned a segment of at least one intron and an exon (Dnja2, Djna3, Psmb7, Mdh2, Ttc38, Eif4h, Aldh1l2, Mcee, Hspg2, and Dyncli2) (Table 3). Four DMRs spanned the first exon as well as the 5′ upstream region (Manf, Canx, Skp1, and Aldh1l1) (Table 3). Ten DMRs were located exclusively in an exon for a given gene (Dtd1, Col6a2, Ppp5c, Arfgap3, Col4a2, Dtdb1, Erp27, Rpn2, Cisd2, and C19gbp) (Table 3). Three DMRs were located in the exons for a gene (Thop1, Dlg1, and Mlec) (Table 3). One DMR was located in the enhancer region for the gene Fisl (Table 3).

### 2.4. Pathway Analyses

Pathway analyses were performed on those targets that were found in both the proteomics and WGBS analyses and had agreeing changes in expression. In the HFD vs. CON female offspring, the pathway analysis identified two targets within the metabolic pathway that exhibited differences in expression (Got2 and Idi1). When evaluating the HFD vs. CON male offspring, our analysis identified five targets that could be classified in the metabolic pathway (Aldh1l1, Bpgm, Mdh2, Rpn2, and Cad). Notably, the gene Rpn2 also featured in the protein processing pathway in addition to Skp1 and Dnaja2. Furthermore, we identified Dctn1 and Dyncil2 within both the vasopressin-regulated water reabsorption and motor processing pathways.

## 3. Discussion

Maternal nutrition plays a vital role in the development of the fetus and it is well established that the negative effects of poor maternal diet can extend into adulthood [7,8,9]. Overnutrition during pregnancy, commonly caused by diets high in sugars and fats, can lead to increased fat deposition as well as impaired β-cell function in the offspring [2,10,20]. In humans, poor nutrition during pregnancy, specifically obesity, has been linked to alterations in the metabolic health of the offspring. Studies show that maternal obesity can predispose children to obesity, insulin resistance, and type 2 diabetes, likely due to the epigenetic changes affecting gene expression related to metabolism [21,22]. There is a critical need to understand how this form of developmental programming can lead to impaired pancreatic function to better understand the origins of diabetes as well as develop effective therapeutics. Therefore, we used an HFD-fed murine model to determine how a Western diet during pregnancy could impact offspring pancreatic tissue DNA methylation and result in corresponding changes to protein expression. This comprehensive, multi-omics approach helped us identify several novel targets for future analyses as well as gain a better understanding of how developmental programming can potentially impair the pancreas and predispose this organ to dysfunction.

Developmental programming research uses a wide variety of animal models including sheep, cattle, swine, rats, and mice [23]. The choice of the model varies depending on the research objectives and the potential applications of the findings [23]. For this study, a mouse model was used because there is a highly annotated reference genome and analytical software available for this species. Furthermore, murine models are more applicable for maternal obesity research than other species [24]. The C57BL/6J mouse strain is a common strain of mouse used in maternal obesity research due to the susceptibility of this mouse line to metabolic disturbances and its propensity to develop obesity when exposed to an HFD [25]. By 8 weeks of age, our female HFD mice weighed more compared to the control animals, which is similar to what others have observed [20,26]. Our animals also followed a trajectory of weight gain that was similar to others. For example, Fang [20] also observed accelerated weight gain in female mice within the initial four weeks of feeding the diet, followed by a plateau around week ten [20]. In general, studies investigating the effects of a Western or high-fat diet during pregnancy typically take one of two approaches. Female mice will remain on the diet prior to conception, throughout pregnancy and lactation. Upon weaning, the pups are maintained on an HFD as well [27,28,29]. Alternatively, other research studies will maintain females on the HFD until parturition, at which point these females are switched to a control diet [30,31,32]. In our study, we adopted a hybridized approach of these commonly used dietary models, where we maintained mothers on an HFD during both pregnancy and lactation and then transitioned all offspring to a control diet after weaning. By maintaining the mice on an HFD during lactation, we aimed to mitigate re-alimentation, which can occur in altricial species (such as mice) during the first 2 weeks postpartum [33]. This could be part of the reason why we did not observe any differences in offspring BW compared with other studies that maintained offspring on high-fat diets after weaning, resulting in an increased BW in these animals. It is also important to note the age of our pups at the time of euthanasia. Mice do not reach sexual maturity until seven weeks of age and mature body size until three months of age [34]. Given that our pups were euthanized shortly after reaching sexual maturity, and did not reach a mature body size, it is possible that they were still too young to exhibit notable differences in weight based on the treatment. Animals that have reached maturity are more likely to create adipose tissue as they are no longer using energy for growth [27]. Future studies that evaluate pup weights at later time points using a model similar to ours may provide additional insight into the effects of the diet.

It has been observed in other species as well as murine models that maternal overnutrition during pregnancy can result in changes to organ development, function, and metabolism in the absence of body weight changes [6,23,35]. This was the case with the present study, where despite not observing any changes in offspring body weight, we were able to identify robust changes in DNA methylation that corresponded with changes in protein expression. Therefore, this subset of corresponding DMRs and proteins are likely factors that deserve additional attention and investigation. This is the purpose of using an multi-omics approach [36]. DMRs can be located in relation to the various DNA motifs (promoter regions, transcription start sites, introns as well as exons) and can have corresponding effects on gene or protein expression [37]. CpG islands, which in turn are flanked by CpG shores [38], represent a small subset of the genome (only 1–2% of a eukaryotic genome comprises a CpG island) [39]. The proportions of our DMRs that are CpG islands and shores are in agreement with this. Furthermore, Diez-Villanueva [40] identified that the majority of DMRs within their dataset were found within the introns, followed by intergenic DMRs, DMRs within a promoter region, and DMRs within exons being the smallest grouping [40]. In the present study, we were able to determine that approximately half of the DMRs that resulted in changes in protein expression were intragenic and/or involved the promoter region of a gene. Building on these insights, our analysis underscores the pivotal role of epigenetic regulation in mediating gene expression outcomes, especially in developmental contexts. Importantly, intragenic and promoter-associated DMRs highlight genetic control, suggesting a targeted epigenetic mechanism that potentially bridges environmental influences and genetic functionality. This correlation between specific methylation patterns and protein expression levels not only strengthens our understanding of regulatory networks but it also opens a new avenue for investigating the epigenetic underpinnings of developmental programming and its implications on health and disease. Studies like those by Gallou-Kabani [41] and Monk [42] have shown that epigenetic mechanisms such as DNA methylation are not just passive responses to developmental signals but are actively involved in the regulation of genes necessary for development. These modifications can be stable and heritable, or dynamic in response to environmental changes, thereby affecting disease susceptibility throughout an individual’s life. It is important to note that we did identify a subset of DMRs that were associated with non-coding RNA species. Non-coding RNAs, such as microRNAs, are classified as an epigenetic modification [43] and can impact transcript availability and splicing. It has also been established that DNA methylation can also impact the expression of microRNA species [44]. RNA-Seq would need to be performed to determine if these RNA species were impacted by the change in methylation status of the DMRs. However, to draw these conclusions at this point would be largely suppositional.

Research on developmental programming has postulated that there is a relationship between DNA methylation and long-term health outcomes [45,46]. The data that we presented in this article help to solidify the concept that DNA methylation changes are key, driving factors in mediating developmental programming impacts on offspring long-term. The impact of an HFD, which is recognized for its role in driving metabolic shifts and inflammation, clearly is capable of modifying DNA methylation landscapes, therefore increasing the incidence of DMRs observed between the treatment groups. Likewise, it appears as though there are differences between the sexes and their responses to an HFD in utero. We observed this in a previous study using an ovine model, when significant differences were observed in the DNA methylation patterns as well as DMRs identified in fetal lambs gestating in overfed or control-fed dams. Overall, the responses of each sex were different from one another [12]. Such alterations could stem from sex-specific responses to the HFD, underscored by hormonal differences, metabolic diversity, and sex-linked genetic variations between males and females [12]. The differences between the sexes were also highlighted when we performed our pathway analyses.

With the CON vs. HFD females, our pathway analysis was limited due to the short list of proteins that correlated with DMRs. Therefore, the pathway analysis was not as robust as the male treatment comparisons. However, regardless of sex, we were able to determine that the majority of these correlating DMRs and proteins were involved in the metabolic processes of pancreatic cells. This is not surprising as we had found this in one of our previous DNA methylation analyses using sheep as a model [12]. Cellular metabolism is a key aspect of cell health. Impaired metabolic function of cells has been observed in the β-cells of patients with diabetes as well as various pancreatic cancer cells lines [47]. Therefore, the data from our present study as well as the previous study in an ovine model suggest that these alterations in cellular metabolic function are key and deserve additional attention. Furthermore, attention needs to be given to the differences in these potential mechanisms between the sexes.

For example, Got2 is involved in the malate–aspartate shuttle and is needed for proper cellular respiration. Consequently, the knockdown of Got2 impairs key cellular metabolic processes (glycolysis and the TCA cycle) while reducing the cellular proliferation of pancreatic cancer cell lines. However, little is known about how a reduction in Got2 protein expression, like the one we observed in the HFD-born female offspring, could impact healthy β-cells/islets. Another poorly studied protein in healthy pancreatic tissue that was part of the metabolic pathway classification in females was Idi1. The mRNA expression of Idi1 increases in vitro in rat β-cell lines in response to treatment with glucose as well as palmitate [48]. The observed reduction in methylation at a locus associated with Idi1 and the corresponding protein increase in our study occurred in the absence of our HFD female offspring being exposed to these substrates. Therefore, our findings suggest that the observed changes are likely not attributable to diet but rather to a programmed effect on the offspring. Studies need to be performed to better understand how GOT2 and Idi1 function in healthy pancreatic tissue as well as evaluate the gene expression, protein expression, and proliferative abilities of β-cells from HFD-born offspring in vitro.

Additional in vitro and in vivo studies need to be performed on the factors that were identified for the CON vs. HFD male offspring given the limited knowledge of their function in healthy pancreatic tissue and the potential role that they play in developmentally programmed offspring. We do know from the literature that Aldh1L1 is involved in folate metabolism, RPN2 is part of the N-oligosaccharyltransferase complex, and Cad plays an integral role in cellular apoptosis [49]. Interestingly, all of these factors exhibited downregulation in the protein expression in the HFD group, which correlated with increased DNA methylation in these offspring. A reduction in the expression of these factors could impact the overall function and health of the pancreas. It is well established that the pancreas experiences a significant rate of cellular turnover and regeneration, particularly within the islets of Langerhans [50,51]. Therefore, a reduction in these factors could contribute to β-cell dysfunction. However, as mentioned previously, more targeted studies need to be performed before these conclusions can be made.

β-cell dysfunction is a critical step towards impaired pancreatic function and ultimately diabetes. β-cell dysfunction has been exhibited in offspring born to mothers who experienced various conditions during pregnancy, such as maternal obesity, gestational diabetes, and undernutrition [52,53]. As one can see from our pathway analyses, it is plausible that some of the factors that we identified may have contributed to the aberrant metabolic function of cells within the pancreas. Likewise, this could be said about the factors that were identified as being part of the protein pathway when comparing CON vs. HFD male offspring. Skp1 is part of a complex of nine proteins (Skp1–Cul–Fbox ligase complex) that is involved in regulating β-cell survival. RPN2, in addition to its metabolic functions, is involved in ribosomal complex formation. Ribosomes are essential for translating mRNA into proteins, including insulin. Efficient ribosomal complex formation ensures that insulin is synthesized accurately and efficiently to meet physiological demands [54]. Dysregulation of RPN2 and its associated pathways can contribute to the development of various pancreatic diseases via defective insulin production, impacting glucose homeostasis [55,56]. Functional studies in healthy β-cells, acinar cells, and cells from developmentally programmed offspring will help to better understand the impact of these factors on pancreatic tissue.

Other factors that may not have matched for a particular pathway also suggest some potential mechanisms by which pancreatic cell function may be compromised. For example, Dctn1 is involved in exocytosis [57] as well as the intracellular transport of insulin-containing vesicles in β-cells [58]. Therefore, dysfunction of this protein or changes in its expression can impair insulin secretion [58]. Increased Dctn1 mRNA expression has been observed in the islets of obese mice (New Zealand obese mice) [57] and increased protein expression of Dctn1 is considered to be a novel biomarker for diabetes [59]. Increased protein expression of Dctn1 was observed in our HFD male animals, suggesting that this may be a potential mechanism by which the dam diet during pregnancy can influence changes in offspring insulin secretion or that this factor deserves additional attention and should be evaluated as a biomarker for the effects of developmental programming. Other factors that help mediate insulin secretion were also identified in our analyses (i.e., F1S1, NSF, Mdh2, and Thop). For example, FIS1 plays a role in regulating mitochondrial fission (i.e., mitochondrial division) and therefore is critical for the proper life cycle of the mitochondria [60]. Reduced Fis1 expression is associated with a reduction in glucose-stimulated insulin release due to the reduced respiratory capacity of the mitochondria [60]. As we observed a reduction in Fis1 in HFD male offspring, it is possible that this could be one of the mechanisms by which hyperglycemia is observed in developmentally programmed offspring [61]. Nsf is thought to play a role in insulin secretion of rats, as it is involved in intracellular transport [62]. Typically, a reduction in Mdh2 expression is observed in individuals with diabetes [63] as it can impact insulin release, and Mdh2 mutations have been observed in families that exhibit transgenerational hyperglycemia [63]. Thop1 is an enzyme involved in the degradation of various peptides, including those involved in insulin regulation [64]. Nsf, Mdh2, and Thop1 protein expression were all reduced in HFD males coupled with increased DNA methylation, thus solidifying the link that these factors may have in mediating some of the effects of developmental programming in offspring born to HFD-fed dams.

When considering the different proteins and DMRs that we identified within our analyses, it is important to remember that we performed our analyses on whole pancreatic tissue. As a result, some of these proteins could have played a role in exocrine tissue function. One such protein is Fabp5. Reduced protein expression of Fabp5 has been observed in pancreatic stellate cells in diseased states such as pancreatitis and ductal adenocarcinomas when compared with healthy fibroblasts [65]. This expression pattern is similar to what we observed in our HFD male mice when compared with CON. Therefore, it is likely that maternal HFD feeding impacted cell types aside from the β-cells of the pancreas and that this could have contributed to diminished pancreatic health and function in the offspring. Additional work needs to be performed to evaluate how pancreatic stellate cells are impacted by developmental programming and how this may contribute to pancreatic dysfunction.

Erp27 is crucial for maintaining pancreatic β-cell homeostasis by aiding in the proper folding of proteins within the endoplasmic reticulum [66]. Klyosova [66] highlighted the unique affinity of Erp27 for unfolding proteins, suggesting its role in attracting protein disulfide isomerase, which is key for maintaining protein homeostasis in β-cells. This protein folding capability is critical in preventing ER stress which can lead to β-cell dysfunction, apoptosis, and ultimately, diabetes. Because we observed a reduction in this factor in HFD male offspring, this could be a mechanism that could increase β-cell loss. Likewise, Manf plays an integral role in the survival and proliferation of β-cells. Recent data suggest that Manf can protect human pancreatic β-cells from stress-induced apoptosis, which as mentioned previously is linked to diabetes [67]. This observed reduction in Manf protein expression could be another way, in addition to Erp27, by which β-cell dysfunction is promoted in HFD-born males. Research that focuses on elucidating the role of these two factors in developmentally programmed offspring would help in better understanding their potential role in pancreatic health as well as the targets for intervention strategies. As mentioned previously, these future studies need to include cellular apoptosis assays and proliferation assays.

In conclusion, our study used a multi-omics approach to identify changes in DNA methylation which resulted in changes to critical and novel proteins. Our work also helps support that there are distinct, gender-specific alterations in protein expression and DNA methylation in response to exposure to an HFD diet while in utero. Further research is needed to elucidate the consequences of these alterations and investigate the interplay between these factors and other metabolic regulators. This will provide a more comprehensive understanding of the complex metabolic pathways affected by developmental programming.

## 4. Materials and Methods

### 4.1. Animals

All procedures and protocols performed were approved by the University of Rhode Island Institutional Animal Care and Use Committee (Protocol # AN1819-018). Mice were purchased from Jackson Laboratory (Bar Harbor, ME, USA) and shipped to the University of Rhode Island where they were group housed (four per cage) in ventilated cages (Innovive, Billerica, MA, USA) maintained at a temperature of 70°F ± 2 with relative humidity of 20–30%. A standard 12-h light/dark cycle was followed. Five-week-old mice (C57BL/6J strain; n = 42; n = 32 females and n = 10 males) arrived at the University of Rhode Island and were quarantined for one week. Starting weights were taken once quarantine was completed to give a baseline. Following quarantine, six-week-old female mice were divided into two treatment groups and fed ad libitum either a low-fat diet control (n = 16; 13% fat TD.08485; Envigo, Somerset, NJ, USA) or a high-fat diet (HFD) (n = 16:TD.88137 Pellet; Envigo, Somerset, NJ, USA) containing 42% fat and 34% sugar. HFD and CON females were maintained on their diet for fourteen weeks prior to breeding. At ten weeks on the diet, control females were switched from the low-fat control diet to a standard mouse chow (TD.2020x; Envigo, Somerset, NJ, USA) due to significant supply issues that resulted in a severe backorder on our initial diet. Weekly body weights were collected by placing each dam individually on a pharmacy scale. At twenty weeks of age, female mice were individually placed into a control-fed male cage for 24 h for breeding. Females were monitored for signs of estrus via vaginal opening [68] in order to determine when to place them into the male’s cage. Once removed, females were monitored for signs of pregnancy or heat. A successful mating was detected via the presence of a vaginal plug within 24 h after being removed from the male’s cage. Pregnancy was confirmed by a sudden increase in body weight within 10–14 days after mating [68]. If pregnancy was detected, females were separated and individually housed beginning at gestational day eighteen. Dams were maintained on their respective diets throughout pregnancy and lactation, until the pups were weaned. Once weaned, pups were separated by sex and housed in groups of four until eight weeks of age. All pups were maintained on a control chow (TD.2020x; Envigo, Somerset, NJ, USA) until sampling. There were three dams per treatment group utilized in our analysis, with an average litter size of 6 ± 0.57 pups in the CON group and 4 ± 0.88 in the HFD group.

### 4.2. Sample Collection

At eight weeks of age, the pups were humanely euthanized via CO_2_ induction accompanied by cervical dislocation. Pancreatic tissue was collected, targeting the tail end, and was snap frozen in liquid nitrogen immediately upon removal. Tissue samples were then stored at −80 °C until DNA was isolated.

### 4.3. Whole Genome Bisulfite Sequencing and Analyses

DNA was isolated from whole pancreatic tissue using a commercially available kit (Qiagen, Hilden, Germany). Isolations, library preparations, and sequencing were performed at Azenta, LLC (South Plainfield, NJ, USA). Prior to library preparations, DNA sample concentrations were quantified using a Qubit 2.0 Fluorometer (Life Technologies, Carlsbad, CA, USA). Bisulfite conversion was performed with the EZ DNA Methylation Gold kit (Zymo, Irvine, CA, USA). DNA was fragmented by Covaris (Woburn, MA, USA) and library preparation performed using the Accel-NGS Methyl-Seq kit (Swift Biosciences, Ann Arbor, MI, USA). A ~0.5% unmethylated lambda DNA spike-in was used as a bisulfite conversion rate control and for downstream bioinformatics. Sequencing libraries were validated using the Agilent Tapestation 4200 (Agilent Technologies, Palo Alto, CA, USA) and quantitative PCR (Applied Biosystems, Carlsbad, CA, USA). Libraries were then multiplexed and sequenced on the Illumina HiSeq instrument (4000 or equivalent) according to manufacturer’s instructions. The samples were sequenced using a 2 × 150 paired end (PE) configuration.

### 4.4. Mass Spectrometry

Protein was isolated from the pancreatic tissue using the sample preparation method described previously in [69]. Protein concentrations were determined by BCA Rapid Gold Assay (Thermo Fisher Scientific, Waltham, MA, USA) according to the manufacturer’s instructions, including the use of a BioTek Epoch2 absorbance plate reader (BioTek Instruments, Winooski, VT, USA). Absorbance was read at 450 nm. Isolated protein was then sent to the University of Connecticut for proteomics and ultraperformance liquid chromatography and tandem mass spectrometry (UPLC–MS/MS) analysis using a Q Exactive HF for mass spectrometry.

### 4.5. Data and Statistical Analyses

Dam growth weight data were run on a repeated measure in SAS (Bloomington, MN, USA) and the lowest AIC was selected and re-run on a Huynh–Feldt. Significance was defined by *p* ≤ 0.05. Mouse pup body weights were run using a simple proc mixed code in SAS. Significance was defined by *p* ≤ 0.05. Proteomics data analysis was performed with Scaffold (Scaffold Proteome Software, Portland, OR, USA) using a *t*-test with a Benjamini–Hochberg correction for average precursor intensity. We used a threshold of *p* < 0.01 and significance was defined by *p* ≤ 0.054. WGBS sequencing analyses were performed in collaboration with CD genomics (New York City, NY, USA). Raw reads obtained from sequencing were first trimmed and low-quality reads were filtered using Trim Galore and Cutadapt. Genome alignments to the *Mus musculus* reference genome (mm39) were performed using Bismark with HiSat2. Differentially methylated regions and the associated locations were determined using methylKit. Default parameters were used for these analyses. Mapping percentages returned from the Bismark alignment were greater than 49% when analyzed in paired end mode. 

### 4.6. WGBS and Proteomics Comparison

In order to determine if changes to the DNA methylation patterns of the offspring were reflected at the protein level, direct comparisons between the differentially methylated regions identified using Methylkit and the differential methylation proteins identified using Scaffold were performed. Specifically, proteins that had a *p*-value ≤ 0.054 were compared to the DMR that had a q-value ≤ 0.05 using Microsoft Excel (Redmond, WA, USA). If there was overlap, comparisons were made to determine if the changes in expression were reflected in the methylation changes in the DNA. 

## 5. Conclusions

Our study confirmed that there are gender-specific changes in pancreatic DNA methylation as well as protein expression in response to in utero exposure to HFD. Furthermore, HFD supplementation during pregnancy causes long-term changes to the pancreatic tissue DNA methylation that are reflected in the corresponding protein expression. Therefore, changes in DNA methylation are one of the key factors mediating the effects of developmental programming in offspring. We also conclude that the novel factors that we identified could merit further analysis on their role in endocrine and exocrine pancreatic function. We also conclude that additional research needs to be performed on these factors in pancreatic tissue. This includes focusing on acinar cells in pancreatic tissue within developmental programming research as well.

## Figures and Tables

**Table 1:** Dam and pup statistics.

	CON	HFD	*p*-Value
Dam starting weight, g ^1^	17.97 ± 0.16	18.5 ± 0.21	*p* = 0.28
Weight at breeding, g ^2^	23 ± 0.13 ^a^	24.81 ± 0.36 ^b^	*p* = 0.01
Average number of pups ^3^	6 ± 0.57	4 ± 0.88	*p* = 0.44
Average number of male pups ^4^	2.6 ± 0.88	1.6 ± 0.33	*p* = 0.24
Average number of female pups ^5^	2.6 ± 0.57	2.3 ± 1.15	*p* = 0.37
Male pup weight, g ^6^	21.25 ± 1.38	19 ± 0.54	*p* = 0.10
Female pup weight, g ^7^	17.25 ± 0.31	16.43 ± 0.52	*p* = 0.50

Data are represented as the means ± standard deviations, ^1^ Dam body weight measurements at 6 weeks of age (CON n = 16; HFD n = 16); ^2^ Average body weight of female mice after 14 weeks on designated diet (CON n = 16; HFD n = 16); ^3^ Average number of pups per litter (CON n = 16; HFD n = 12); ^4^ Average number of male pups per litter (CON n = 8; HFD n = 5); ^5^ Average number of female pups per litter (CON n = 8; HFD n = 7); ^6^ Body weight of male pups at eight weeks of age; ^7^ Body weight of female pups at eight weeks of age. ^a,b^ Denotes *p* ≤ 0.05 as determined by Student’s *t*-test.

**Table 2:** DNA methylation analyses.

	CON	HFD	Average
Raw Q30% ^1^	96.5	96.4	96.5
Number of Reads post-trim ^2^	8,228,506	8,687,983.3	12,369,237.1
Trimmed Q30% ^3^	97	97	97
% Mapping ^3^	51.06	51.9	51.4

Data are based on averages of the treatment groups. ^1^ Percentage of reads with a q-score greater than 30 prior to trimming and filtering; ^2^ Number of reads post-trim and after filtering with Trim Galore and Cutadapt; ^3^ Percentage of reads mapping to the reference annotation in paired end mode using Bismark. CON n = 16; HFD n = 12.

**Table 3:** HFD vs. CON males average protein abundance and DMR location.

Location	CON Avg. ^1^	HFD Avg. ^2^	Methylation Diff ^3^	Feature Strand ^4^	Feature Symbol
chr6:34471936-34472435	1.1 × 10^7^	0.0 × 10^0^	14.25	+	Bpgm *
chr11:103710513-103711012	1.5 × 10^7^	6.0 × 10^6^	11.18	−	Nsf *
chr16:4457865-4458364	6.5 × 10^6^	0.0 × 10^0^	7.05	+	Dnaja3 *
chr5:135807985-135808484	1.0 × 10^8^	3.0 × 10^7^	3.95	+	Mdh2 *
chr5:136970985-136971484	6.2 × 10^6^	2.8 × 10^1^	9.34	+	Fis1 *
chr8:84441231-84441730	1.8 × 10^7^	3.1 × 10^6^	6.02	+	Ddx39a *
chr11:52122826-52123325	4.35 × 10^6^	0.0 × 10^0^	1.74	+	Skp1 *
chr2:144521685-144522184	1.74 × 10^7^	8.4 × 10^6^	17.06	+	Dtd1 *
chr4:137286397-137286896	5.5 × 10^6^	1.1 × 10^7^	−9.46	+	Hspg2 *
chr6:90526989-90527488	2.9 × 10^7^	1.1 × 10^7^	16.99	+	Aldh1l1 *
chr16:31690233-31690732	6.5 × 10^6^	0.0 × 10^0^	4.71	+	Dlg1 *
chr9:17899853-17900352	7.0 × 10^6^	0.0 × 10^0^	−15.08	+	Chordc1
chr7:64049863-64050362	4.8 × 10^6^	0.0 × 10^0^	10.54	+	Mcee *
chr7:117696210-117696709	1.2 × 10^8^	2.9 × 10^7^	7.47	−	Rps15a *
chr15:83188272-83188771	1.7 × 10^7^	1.5 × 10^6^	13	−	Arfgap3 *
chr6:83138489-83138988	7.2 × 10^6^	2.8 × 10^1^	3.73	+	Dctn1 *
chr5:31211472-31211971	8.0 × 10^0^	2.1 × 10^6^	6.8	+	Cad *
chr2:71081288-71081787	1.0 × 10^7^	1.6 × 10^6^	9.39	+	Dync1i2 *
chr2:157130685-157131184	6.6 × 10^7^	1.3 × 10^7^	8.06	+	Rpn2 *
chr5:134674485-134674984	2.3 × 10^7^	2.0 × 10^6^	12.02	−	Eif4h *
chr8:86281731-86282230	1.1 × 10^7^	2.1 × 10^6^	2.03	−	Dnaja2 *
chr10:76453090-76453589	4.7 × 10^6^	1.5 × 10^7^	−2.64	−	Col6a2 *
chr10:80916090-80916589	2.3 × 10^7^	6.7 × 10^6^	3.61	+	Thop1 *
chr5:115283051-115283550	4.8 × 10^7^	1.5 × 10^7^	5.71	−	Mlec *
chr3:135120798-135121297	3.9 × 10^7^	1.4 × 10^7^	6.62	−	Cisd2 *
chr3:10033561-10034060	2.4 × 10^7^	8.7 × 10^6^	8.19	+	Fabp5 *
chr11:70871325-70871824	3.7 × 10^7^	5.9 × 10^6^	8.43	−	C1qbp *
chr6:136895478-136895977	9.0 × 10^7^	2.0 × 10^7^	37.27	−	Erp27 *
chr10:83342620-83343119	4.6 × 10^7^	1.3 × 10^7^	13.69	−	Aldh1l2 *
chr2:38533993-38534492	1.1 × 10^7^	0.0 × 10^0^	7.97	−	Psmb7 *
chr9:106767369-106767868	9.5 × 10^7^	2.6 × 10^7^	27.22	−	Manf *
chr7:16760077-16760576	1.5 × 10^7^	7.0 × 10^6^	16.84	−	Ppp5c *
chr11:50216326-50216825	7.0 × 10^7^	3.4 × 10^7^	2.79	−	Canx *
chr15:85716772-85717271	1.2 × 10^7^	2.8 × 10^6^	13.28	+	Ttc38 *
chr14:30263272-30263771	6.0 × 10^7^	2.0 × 10^7^	24.04	+	Tkt *
chr8:11329001-11329500	2.1 × 10^6^	1.8 × 10^7^	−8.58	+	Col4a2 *

^1^ Average relative protein abundance for CON (n = 8) males; ^2^ Average relative protein abundance for HFD (n = 5) males; ^3^ Denotes the fold change relative to CON for DNA methylation analyses; ^4^ Identifies if the DMR was located on the sense (+) or antisense (−) strand of the DNA. Biphosphoglycerate mutase (Bpgm); N-ethylmaleimide-sensitive fusion protein (Nsf); DnaJ heat shock protein family (Dnaja) member 3; Malate dehydrogenase (Mdh) 2; Fission mitochondrial (Fis) 1; DEAD box helicase (Ddx) 39a; S-phase kinase-associated protein (Skp) 1; D-tyrosyl-tRNA deacylase (Dtd) 1; Perlecan heparan sulfate proteoglycan (Hspg) 2; Aldehyde dehydrogenase (Aldh) family 1 member L1; Discs large MAGUK scaffold protein (Dlg) 1; Cysteine- and histidine-rich domain (Chordc) 1; Methylmalonyl CoA epimerase (Mcee); Ribosomal protein S (Rps) 15a; ADP ribosylation factor GTPase activating protein (Arfgap) 3; Dynactin (Dctn) 1; Carbamoyl-phosphate synthetase (Cad); Dynein cytoplasmic (Dync) 1 intermediate chain 2; Ribophorin (Rpn) 2; Eukaryotic translation inhibition factor (Eif) 4h; DnaJ heat shock protein family (Dnaja) member 2; Collagen (Col) type 6 alpha 2; Thimet oligopeptidase (Thop) 1; Malectin (Mlec); CDGSH iron sulfur domain (Cisd) 2; Fatty acid-binding protein (Fabp) 5; Complement component 1 subcomponent-binding protein (Cqpb); Endoplasmic reticulum protein (Erp) 27; Aldehyde dehydrogenase (Aldh) family 1 member L2; Proteasome macropain (Psmb) 7; Mesencephalic astrocyte-derived neurotrophic factor (Manf); Protein phosphatase (Ppp) 5 catalytic subunit; Calnexin (Canx); Tetratricopeptide (Ttc) 38; Transketolase (Tkt); Collagen (Col) type 4 alpha 2. * Denotes proteins that exhibited a change in expression that agreed with the change observed in DNA methylation (e.g., reduction in protein expression was associated with increased DNA methylation at a specific DMR). Data were determined using WGBS and LCMS analyses.

**Table 4:** HFD vs. CON female average protein abundance and DMR location.

Location	CON Avg. ^1^	HFD Avg. ^2^	Methylation Diff ^3^	Feature Strand ^4^	Feature Symbol
chr2:164674421-164674920	1.71 × 10^7^	7.38 × 10^6^	8.37	+	Ctsa *
chr12:113284910-113285409	1.21 × 10^7^	2.38 × 10^7^	−11.19	−	Ighg1 *
chr13:8935791-8936290	1.53 × 10^6^	5.93 × 10^6^	−1.39	+	Idi1 *
chr8:96594729-96595228	2.15 × 10^7^	5.66 × 10^7^	−6.01	−	Got2 *

^1^ Average relative protein abundance for CON (n = 8) females; ^2^ Average relative protein abundance for HFD (n = 7) females; ^3^ Denotes the fold change relative to CON for DNA methylation analyses; ^4^ Identifies if the DMR was located on the sense (+) or antisense (−) strand of the DNA. Cathepsin A (Ctsa); Immunoglobulin heavy constant gamma (Ighg) 1; Carbonyl reductase (Cbr) 1; Isopentenyl-diphosphate delta isomerase (Idi) 1; Inositol-3-phosphate synthase (Isyna) 1; Glutamic-oxaloacetic transaminase (Got) 2. * Denotes proteins that exhibited a change in expression that agreed with the change observed in DNA methylation (e.g., reduction in protein expression was associated with increased DNA methylation at a specific DMR). Data were determined using WGBS and LCMS analyses.

## Data Availability

The raw data used in this study can be made available upon reasonable request via the corresponding author.
